# 4D flow yields similar clinical results compared to 2D phase contrast for decision making regarding pulmonary valve replacement in repaired tetralogy of Fallot

**DOI:** 10.21203/rs.3.rs-6280623/v1

**Published:** 2025-04-16

**Authors:** Alison Almgren-Bell, Andrada Popescu, Aparna Sodhi, Michael Markl, Cynthia Rigsby, Joshua Robinson

**Affiliations:** Northwestern University; Lurie Children’s Hospital; Lurie Children’s Hospital; Northwestern University; Lurie Children’s Hospital; Lurie Children’s Hospital

**Keywords:** cardiac MRI, 4D flow, Tetralogy of Fallot, pulmonary valve replacement

## Abstract

2D CMR is critical for monitoring PR fraction (PRF) and biventricular size in patients with a history of repaired tetralogy of Fallot and guides decisions about pulmonary valve replacement (PVR). However, its clinical utility is limited, increasing enthusiasm for newer techniques such as 3D time-resolved phase contrast (4D flow) MRI. We investigated whether using 4D flow to calculate PRF would yield similar clinical decisions about PVR in rTOF compared to conventional 2D CMR. All patients with rTOF who underwent standard CMR plus retrospectively gated 4D flow between February 2021 – June 2023 were identified. Clinical information was collected from the EMR. 2D cine SSFP, phase contrast (PC) data, and 4D flow imaging data were analyzed using standard post-processing analysis. Clinical decisions (“intervention vs. no intervention”) were determined using a standardized algorithm, and inter-rater agreement was assessed using the coefficient. 33 patients were included. PRF correlated strongly between 2D PC and 4D flow (r=0.83) and with PRF determined by stroke volume (r=0.70). The mean absolute difference between 2D and 4D PRF was −3.4% (± 9.3%). Inter-rater agreement for the decision was moderate (=0.58) using only 2D PC and 4D flow, and was strong (=0.76) when using 2D PC with SV and 4D flow with SV. Though clinical indications for PVR in rTOF vary, 4D flow generates accurate measurements of PRF and yields similar clinical decisions about intervention in rTOF. Further study of ventricular volume assessment by 4D flow adoption could lead to a shorter, more comprehensive CMR exam for TOF patients.

## INTRODUCTION

Tetralogy of Fallot (TOF) is the most common cyanotic congenital heart defect (CHD) with an estimated prevalence of 4.6/10,000 new cases per year [1–3]. While surgical outcomes and long-term prognosis have improved significantly, residual anatomic and hemodynamic abnormalities are common [3–6]. Chronic pulmonary regurgitation (PR) is a common hemodynamic complication following initial repair of TOF and frequently leads to right ventricular (RV) dilation and dysfunction with increased risk for exercise intolerance, right heart failure, arrhythmias, and sudden death [3, 7–10] Pulmonary valve replacement (PVR) reduces PR, decreases RV overload, and normalizes RV volumes [4, 11]. However, the hemodynamic benefits of PVR must be balanced with the risks of premature invention and surgical complications including valve failure and death [4]. These considerations are in uenced by growing transcatheter PVR options such that optimal timing of PVR remains highly debated. The decision to pursue intervention involves careful consideration of natural history and pathophysiology as well as procedural risks and benefits [4, 7, 11, 12].

2D cardiac magnetic resonance (CMR) is currently the gold standard for monitoring PR, ventricular function, and ventricular volumes in patients with repaired TOF (rTOF) [4, 13, 14]. However, long scan times (often >60 minutes) and breath-holding requirements present challenges to clinical work flow and widespread usage, especially in younger patients and those with developmental delay [10, 13, 15]. 3D time-resolved phase contrast (4D flow) CMR is a novel technique that allows magnitude and phase contrast (PC) data to be obtained from a single, free-breathing sequence [13, 16]. 4D flow scan time is an average of 5–15 minutes and thus may decrease the need for anesthesia in younger patients as well as patients with decompensated heart failure or developmental delay, making it a promising new technique for long-term monitoring of rTOF patients [5, 13, 16].

Several studies have shown that 4D flow performs similarly to 2D PC in measurements of blood flow and ventricular volume [13, 17, 18]. In one study of rTOF patients, 4D flow demonstrated greater accuracy compared to 2D PC for flow measurements and performed similarly to 2D SSFP for volumetric analysis [13]. While agreement between 2D and 4D flow is established, the implications of 4D flow on real-world PVR decision making have not been fully studied [13]. Moreover, the growing availability of commercial software applications has made 4D flow post-processing easier, less time consuming and more clinically feasible. This study is a retrospective comparison of clinical decision making in the management of rTOF with 2D PC CMR versus 4D flow to test the hypothesis that 4D flow provides accurate clinical information that yields similar clinical decisions.

## MATERIALS AND METHODS

### Study population

We retrospectively identified all patients with rTOF who underwent standard clinical CMR with cine SSFP and PC imaging between February 2021 and June 2023 at Lurie Children’s Hospital. The medical center IRB approved this study, and informed consent was waived for retrospective clinical data collection. Patients who had undergone prior PVR were included. Patients who did not have diagnostic quality CMR exams, who had important metallic artifact, and who did not have both 2D phase contrast and 4D flow imaging were excluded. Demographic data and clinical history were manually extracted from the electronic medical record and included patient age, sex, BSA, cardiac diagnoses and number of prior cardiac intervention(s), symptoms, and EKG results including QRS duration.

### Image acquisition

Imaging was performed on a 1.5T scanner (MAGNETOM Aera; Siemens Healthineers, Erlangen, Germany) using an 18-channel matrix coil. Standard balanced steady-state free precession cine imaging and 2-D PC scans were obtained per routine clinical protocol and as appropriate for the assessment of patients with TOF [19]. If contrast was administered, 2D PC MRI and 4D flow were acquired following administration of either ferumoxytol (Feraheme, AMAG Pharmaceuticals, Waltham, MA) or gadobutrol (Gadavist, Bayer HealthCare, Whippany, NJ). 4D flow scan characteristics for this cohort were: spatial resolution = 1.4–4.5 × 1.1–3.1 × 1.4–3.5 mm^3^, temporal resolution = 36.0–44.8 ms, repetition time/echo time [TR/TE] = 4.7–5.1/2.2–3.0 ms, flip angle = 15°, and velocity sensitivity (VENC) = 100–250 cm/s. General anesthesia was administered by a pediatric anesthesiologist per the institutional clinical protocol for 9/33 patients (27%).

### Image analysis

**Figure 1:**
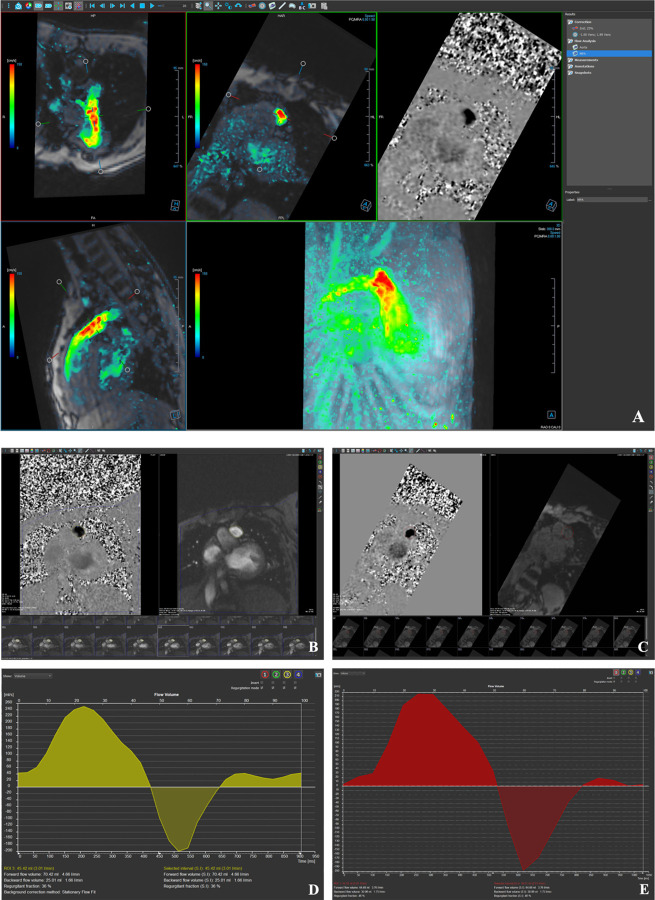
(A) Example of 4D flow visualization and quantification with pulmonary artery plane placement. ROI placement for 2D PC (B) and 4D flow (C). Flow-time curves of PR using 2D PC (D) and 4D (E).

2D SSFP and PC data were obtained from the clinical CMR report completed by the cardiologist and radiologist. PRF by SV was calculated using the following equation: PRF = (SV_RV_ – SV_LV_) / SV_RV._ Qflow software (Medis Suite, Leiden) was used for 4D flow post processing (Figure 1). Background phase offset corrections were performed, and the phase unwrap tool was applied if there was velocity aliasing For 4D flow analysis, cross-sectional planes were oriented perpendicular to the direction of flow and vessel anatomy. Pulmonary flow was measured at the main pulmonary artery, and the 3D region of interest was manually drawn around the vessel lumen directly above the pulmonary valve. flow parameters such as net flow, forward flow, backward flow, and PRF were quantified and recorded.

### PVR decision-making algorithm

**Figure 2:**
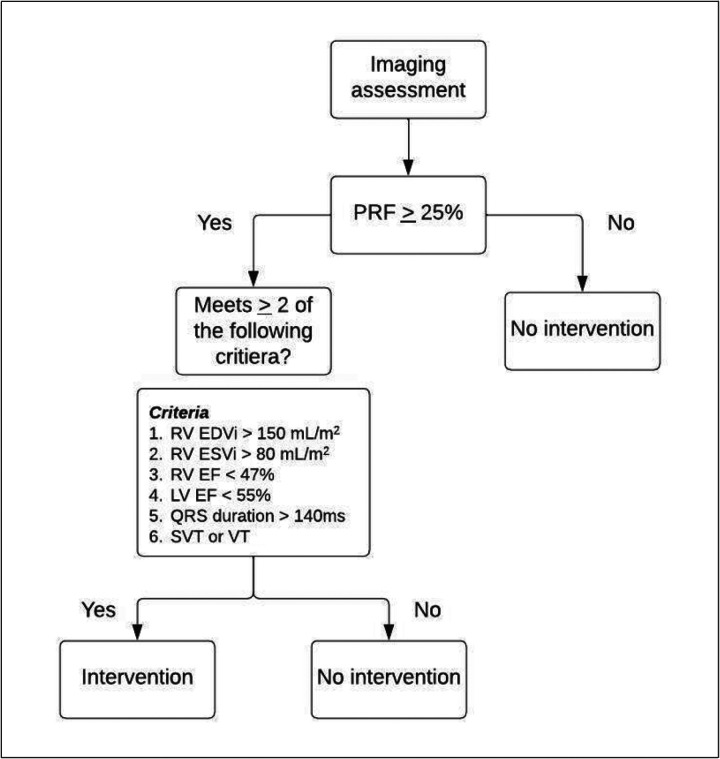
Clinical decision-making algorithm adapted from conventional guidelines [4].

Clinical decisions regarding PVR (“intervention vs. no intervention”) were determined using the conventional indications algorithm outlined in the flow chart Figure 2) [4]. Decisions were first made using the PRF calculated only by 2D PC or 4D flow. The same algorithm was then run again using PRF calculated by 2D PC or 4D flow in conjunction with PRF calculated via SV difference, similar to conventional clinical practice.

### Statistical analysis

All continuous variables were reported as mean ± standard deviation, and categorical variables were reported as percentage and frequency. Bland-Altman analyses were used to assess agreement between different methods. Inter-rater agreement for the decision was measured using Cohen’s kappa coefficient.

## RESULTS

**Figure 3:**
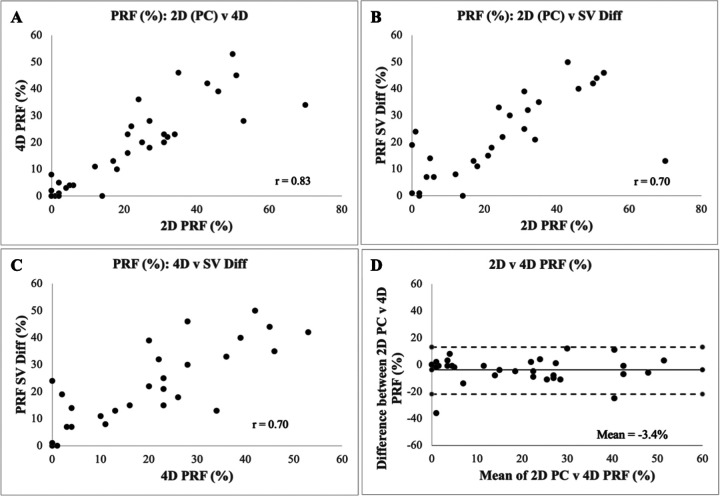
Scatter plots demonstrating correlation between (A) 2D PC and 4D flow (B) 2D PC and SV Diff (C) 4D flow and SV diff. (D) Bland-Altman plot comparing PRF by 2D PC and 4D flow.

Fifty patients met initial criteria during the defined study time period. Fifteen patients were excluded due to lack of 2D (n=6) or 4D (n=9) PC data, and two patients were excluded due to metallic artifact precluding PV assessment, resulting in a study population of 33 patients. Contrast was administered in 15/33 exams (45%). Patient demographics are shown in Table 1. Important differences among groups stratified by clinical decision include RV end-diastolic volume (EDV), RV end-systolic volume (ESV), LV ejection fraction (EF), and PRF measured by 2D PC and 4D flow. PRF calculated by 2D PC correlated strongly with 4D flow-derived PRF (r=0.83) (Figure 3). PRF showed moderate-strong correlation between 2D PC and SV difference (r=0.70) and between 4D flow and SV difference (r=0.70) (Figure 3).

Algorithm-based decisions for PVR are shown in Table 2. 28 decisions (85%) were concordant between 2D PC and 4D flow. Intervention was recommended in 23 of these cases, and no intervention was recommended in the other 5. There were five discordant decisions; in all five, 2D recommended intervention whereas 4D recommended no intervention. When using PRF calculated by 2D only and 4D only, the inter-rater agreement was moderate (*κ* = 0.58). When using PRF calculated by 2D with SV and 4D with SV, there were three discordiant decisions, in which 2D recommended intervention whereas 4D recommended no intervention. The inter-rater agreement improved and was strong (*κ* = 0.76).

## DISCUSSION

In this study, we assessed the utility of 4D flow compared to 2D PC in real-world clinical decision-making regarding indication for PVR in the rTOF population. We also compared PRF measured by 4D flow, 2D PC, and SV difference and found strong correlations between PRF measured via all three methods. These findings are consistent with prior studies that similarly found robust agreement between flow measurements obtained via 2D PC and 4D flow [17, 18]. We also found that 4D flow and 2D PC resulted in similar clinical decisions about recommendation for PVR using a conventional algorithm. Using all available information (both flow and cine SSFP derived) resulted in nearly universal agreement. Based on these findings, 4D flow holds significant promise in the evaluation and management of patients with rTOF.

This study offers novel insight into the role of 4D flow in conventional decision-making for patients with rTOF. Despite several studies investigating ideal indications for intervention, the decision surrounding intervention remains highly nuanced and individualized with significant practice variation across centers and providers [20, 21]. Several studies have found that intervention in patients satisfying volumetric requirements leads to improvement in RV volume and function, yet the specific thresholds predictive of optimal outcome vary across studies [22–26]. Massarella et al examined referral patterns in a multicenter cohort of rTOF patients and found that majority of their cohort met criteria for PVR yet were not referred for intervention [20]. Further analysis of their cohort suggested that thresholds for referral proposed in guidelines are more aggressive than the thresholds used to guide decision-making in everyday clinical practice [20]. The small differences in agreement across clinical decisions found in our study fall well within the range of practice variation.

In addition to 4D flow’s strong clinical performance in determining the severity of PR and indication for PVR, this technique offers additional insights into higher order fluid dynamics and evolving hemodynamic efficiencies [5]. Physiological parameters derived from 4D flow like kinetic energy (KE), helix and vortex formation, energy loss, and wall shear stress have shown promise as early biomarkers in TOF [27–35]. RV KE has been shown to be higher in TOF patients compared to healthy controls and correlate with volumetric and flow markers of disease severity, including RV EDVi and PRF [27, 30–32]. Altered hemodynamic forces and flow patterns, including pathologic vortex formation, are seen in TOF patients and have been shown to correlate with RV dysfunction and exercise intolerance [28, 33–35].

In summary, the ability to capture conventional and novel hemodynamic parameters in under ten minutes with a single, free-breathing, scan combined with the recent advancements in commercially available post-processing software makes 4D flow an increasingly feasible and compelling addition to the current clinical workflow for management of patients with rTOF [36].

Limitations of this study include our small study cohort of patients with rTOF and retrospective study design. Another limitation of this study is the focus on agreement between 2D PC and 4D flow; future study of ventricular volume assessment by 4D flow or in combination with other accelerated techniques could lead to an even shorter, comprehensive exam for TOF patients.

## Figures and Tables

**Table 1:** Demographic, clinical, and radiographic information of repaired tetralogy of Fallot patients stratified based on history of prior PVR and clinical decision regarding PVR. P values calculated using ANOVA or chi square with * indicating significance (p < 0.05).

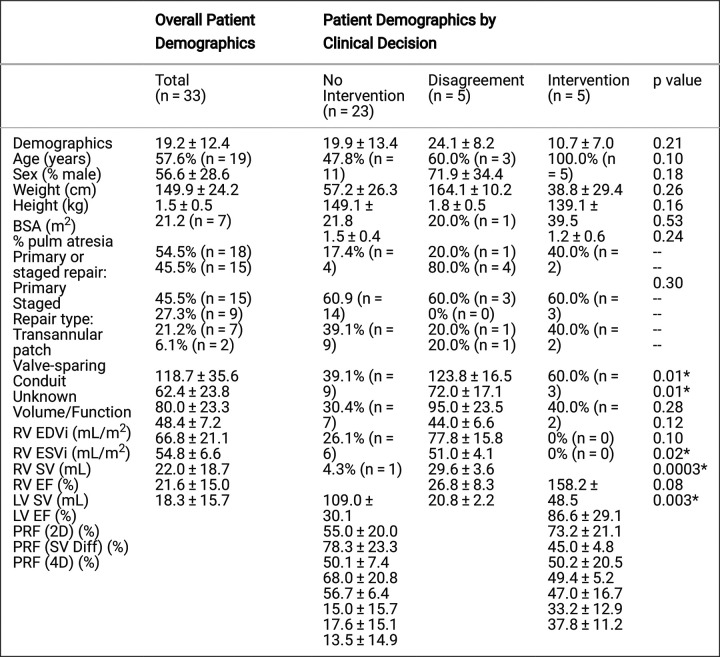

**Table 2:** Top pane shows clinical decisions made using the PRF calculated only by 2D PC or 4D flow. Bottom pane shows decisions made using the same algorithm but with PRF calculated by 2D PC or 4D flow in conjunction with PRF calculated via stroke volume difference.

		4D		Inter-rater agreement (K): 0.58
		No intervention	Intervention	
2D	No intervention	23	0	
	Intervention	5	5	
		4D + SV		Inter-rater agreement (K): 0.76
		No intervention	Intervention	
2D + SV	No intervention	23	0	
	Intervention	3	7	

## Abbreviations

CHD: congenital heart disease
CMR: cardiac magnetic resonance
LV: left ventricle
RV: right ventricle
SV: stroke volume
TOF: tetralogy of Fallot
PC: phase contrast
PR: pulmonary regurgitation
PRF: pulmonary regurgitant fraction
PVR: pulmonary valve replacement
rTOF: repaired tetralogy of Fallot

## References

[1] Apitz C, Webb GD, Redington AN (2009) Tetralogy Fallot Lancet 374(9699):1462–1471 19683809 10.1016/S0140-6736(09)60657-7

[2] Mai CT (2019) National population-based estimates for major birth defects, 2010–2014. Birth Defects Res 111(18):1420–1435 31580536 10.1002/bdr2.1589 PMC7203968

[3] Gatzoulis MA (2000) Risk factors for arrhythmia and sudden cardiac death late after repair of tetralogy of Fallot: a multicentre study. Lancet 356(9234):975–981 11041398 10.1016/S0140-6736(00)02714-8

[4] Geva T (2011) Repaired tetralogy of Fallot: the roles of cardiovascular magnetic resonance in evaluating pathophysiology and for pulmonary valve replacement decision support. J Cardiovasc Magn Reson 13(1):9 21251297 10.1186/1532-429X-13-9 PMC3036629

[5] Elsayed A (2021) Four-dimensional flow cardiovascular magnetic resonance in tetralogy of Fallot: a systematic review. J Cardiovasc Magn Reson 23(1):59 34011372 10.1186/s12968-021-00745-0 PMC8136126

[6] Nollert G (1997) Long-term survival in patients with repair of tetralogy of Fallot: 36-year follow-up of 490 survivors of the first year after surgical repair. J Am Coll Cardiol 30(5):1374–1383 9350942 10.1016/s0735-1097(97)00318-5

[7] Flors L (2020) Preprocedural Imaging Evaluation of Pulmonary Valve Replacement After Repair of Tetralogy of Fallot: What the Radiologist Needs to Know. J Thorac Imaging 35(3):153–166 32073541 10.1097/RTI.0000000000000478

[8] House AV (2019) Can Abbreviated Cardiac Magnetic Resonance Imaging Adequately Support Clinical Decision Making After Repair of Tetralogy of Fallot? Pediatr Cardiol 40(3):616–622 30539240 10.1007/s00246-018-2035-0

[9] Bissell MM (2023) 4D flow cardiovascular magnetic resonance consensus statement: 2023 update. J Cardiovasc Magn Reson 25(1):40 37474977 10.1186/s12968-023-00942-z PMC10357639

[10] Warmerdam E (2020) Three-dimensional and four-dimensional flow assessment in congenital heart disease. Heart 106(6):421–426 31857355 10.1136/heartjnl-2019-315797

[11] Tatewaki H, Shiose A (2018) Pulmonary valve replacement after repaired Tetralogy of Fallot. Gen Thorac Cardiovasc Surg 66(9):509–515 29779123 10.1007/s11748-018-0931-0

[12] de Torres-Alba F, Kaleschke G, Baumgartner H (2018) Impact of Percutaneous Pulmonary Valve Implantation on the Timing of Reintervention for Right Ventricular Outflow Tract Dysfunction. Rev Esp Cardiol (Engl Ed) 71(10):838–846 29859895 10.1016/j.rec.2018.05.001

[13] Jacobs KG (2020) 4D flow vs. 2D cardiac MRI for the evaluation of pulmonary regurgitation and ventricular volume in repaired tetralogy of Fallot: a retrospective case control study. Int J Cardiovasc Imaging 36(4):657–669 31894524 10.1007/s10554-019-01751-1 PMC8018898

[14] Fratz S (2013) Guidelines and protocols for cardiovascular magnetic resonance in children and adults with congenital heart disease: SCMR expert consensus group on congenital heart disease. J Cardiovasc Magn Reson 15(1):51 23763839 10.1186/1532-429X-15-51 PMC3686659

[15] Oosterhof T (2006) Cardiovascular magnetic resonance in the follow-up of patients with corrected tetralogy of Fallot: a review. Am Heart J 151(2):265–272 16442887 10.1016/j.ahj.2005.03.058

[16] Dyverfeldt P (2015) 4D flow cardiovascular magnetic resonance consensus statement. J Cardiovasc Magn Reson 17(1):72 26257141 10.1186/s12968-015-0174-5 PMC4530492

[17] Gabbour M (2015) 4-D flow magnetic resonance imaging: blood flow quantification compared to 2-D phase-contrast magnetic resonance imaging and Doppler echocardiography. Pediatr Radiol 45(6):804–813 25487721 10.1007/s00247-014-3246-z PMC4450116

[18] Nordmeyer S (2010) Flow-sensitive four-dimensional cine magnetic resonance imaging for offline blood flow quantification in multiple vessels: a validation study. J Magn Reson Imaging 32(3):677–683 20815066 10.1002/jmri.22280

[19] Fogel MA (2022) Society for Cardiovascular Magnetic Resonance/European Society of Cardiovascular Imaging/American Society of Echocardiography/Society for Pediatric Radiology/North American Society for Cardiovascular Imaging Guidelines for the use of cardiovascular magnetic resonance in pediatric congenital and acquired heart disease: Endorsed by The American Heart Association. J Cardiovasc Magn Reson 24(1):37 35725473 10.1186/s12968-022-00843-7 PMC9210755

[20] Massarella D (2024) Adherence to clinical practice guidelines for pulmonary valve intervention after tetralogy of Fallot repair: A nationwide cohort study. JTCVS Open 17:215–228 38420530 10.1016/j.xjon.2023.11.013 PMC10897679

[21] House AV (2015) Impact of clinical follow-up and diagnostic testing on intervention for tetralogy of Fallot. Open Heart 2(1):e000185 25973212 10.1136/openhrt-2014-000185 PMC4422920

[22] Holmes KW (2012) Timing of pulmonary valve replacement in tetralogy of fallot using cardiac magnetic resonance imaging: an evolving process, in J Am Coll Cardiol. : United States. pp. 1015–7 10.1016/j.jacc.2012.05.026 22921970

[23] Geva T (2010) Randomized trial of pulmonary valve replacement with and without right ventricular remodeling surgery. Circulation 122(11 Suppl):S201–S208 20837914 10.1161/CIRCULATIONAHA.110.951178 PMC2943672

[24] Therrien J (2005) Optimal timing for pulmonary valve replacement in adults after tetralogy of Fallot repair. Am J Cardiol 95(6):779–782 15757612 10.1016/j.amjcard.2004.11.037

[25] Oosterhof T (2007) Preoperative thresholds for pulmonary valve replacement in patients with corrected tetralogy of Fallot using cardiovascular magnetic resonance. Circulation 116(5):545–551 17620511 10.1161/CIRCULATIONAHA.106.659664

[26] Buechel ER (2005) Remodelling of the right ventricle after early pulmonary valve replacement in children with repaired tetralogy of Fallot: assessment by cardiovascular magnetic resonance. Eur Heart J 26(24):2721–2727 16214832 10.1093/eurheartj/ehi581

[27] Robinson JD (2019) 4-D flow magnetic-resonance-imaging-derived energetic biomarkers are abnormal in children with repaired tetralogy of Fallot and associated with disease severity. Pediatr Radiol 49(3):308–317 30506329 10.1007/s00247-018-4312-8 PMC6382568

[28] Loke YH (2021) Moving beyond size: vorticity and energy loss are correlated with right ventricular dysfunction and exercise intolerance in repaired Tetralogy of Fallot. J Cardiovasc Magn Reson 23(1):98 34412634 10.1186/s12968-021-00789-2 PMC8377822

[29] Elsayed A (2021) Right Ventricular flow Vorticity Relationships With Biventricular Shape in Adult Tetralogy of Fallot. Front Cardiovasc Med 8:806107 35127866 10.3389/fcvm.2021.806107 PMC8813860

[30] Fredriksson A (2018) Turbulent kinetic energy in the right ventricle: Potential MR marker for risk stratification of adults with repaired Tetralogy of Fallot. J Magn Reson Imaging 47(4):1043–1053 28766919 10.1002/jmri.25830

[31] Jeong D (2015) Ventricular kinetic energy may provide a novel noninvasive way to assess ventricular performance in patients with repaired tetralogy of Fallot. J Thorac Cardiovasc Surg 149(5):1339–1347 25623907 10.1016/j.jtcvs.2014.11.085 PMC4437857

[32] Sjöberg P (2018) Disturbed left and right ventricular kinetic energy in patients with repaired tetralogy of Fallot: pathophysiological insights using 4D-flow MRI. Eur Radiol 28(10):4066–4076 29666995 10.1007/s00330-018-5385-3 PMC6132722

[33] Sjöberg P (2018) Altered biventricular hemodynamic forces in patients with repaired tetralogy of Fallot and right ventricular volume overload because of pulmonary regurgitation. Am J Physiol Heart Circ Physiol 315(6):H1691–h1702 30265559 10.1152/ajpheart.00330.2018

[34] François CJ (2012) 4D cardiovascular magnetic resonance velocity mapping of alterations of right heart flow patterns and main pulmonary artery hemodynamics in tetralogy of Fallot. J Cardiovasc Magn Reson 14(1):16 22313680 10.1186/1532-429X-14-16 PMC3305663

[35] Geiger J (2011) 4D-MR flow analysis in patients after repair for tetralogy of Fallot. Eur Radiol 21(8):1651–1657 21720942 10.1007/s00330-011-2108-4

[36] Takehara Y, Sekine T, Obata T (2022) Why 4D flow MRI? Real Advantages. Magn Reson Med Sci 21(2):253–256 35197415 10.2463/mrms.e.2022-1000 PMC9680543

